# Continuous Acquisition of MHC:Peptide Complexes by Recipient Cells Contributes to the Generation of Anti‐Graft CD8^+^ T Cell Immunity

**DOI:** 10.1111/ajt.13996

**Published:** 2016-09-14

**Authors:** L. A. Smyth, R. I. Lechler, G. Lombardi

**Affiliations:** ^1^ Medical Research Council (MRC) Centre for TransplantationKing's College LondonLondonUK; ^2^ National Institute for Health Research (NIHR) Comprehensive Biomedical Research CentreGuy's and St. Thomas’ NHS Foundation Trust and King's College LondonLondonUK; ^3^ School of Health, Sport and BioscienceUniversity of East LondonLondonUK

**Keywords:** basic (laboratory) research/science, immunosuppression/immune modulation, immunobiology, histocompatibility, immune regulation, cytotoxicity, innate immunity

## Abstract

Understanding the evolution of the direct and indirect pathways of allorecognition following tissue transplantation is essential in the design of tolerance‐promoting protocols. On the basis that donor bone marrow–derived antigen‐presenting cells are eliminated within days of transplantation, it has been argued that the indirect response represents the major threat to long‐term transplant survival, and is consequently the key target for regulation. However, the detection of MHC transfer between cells, and particularly the capture of MHC:peptide complexes by dendritic cells (DCs), led us to propose a third, semidirect, pathway of MHC allorecognition. Persistence of this pathway would lead to sustained activation of direct‐pathway T cells, arguably persisting for the life of the transplant. In this study, we focused on the contribution of acquired MHC‐class I on recipient DCs during the life span of a skin graft. We observed that MHC‐class I acquisition by recipient DCs occurs for at least 1 month following transplantation and may be the main source of alloantigen that drives CD8^+^ cytotoxic T cell responses. In addition, acquired MHC‐class I:peptide complexes stimulate T cell responses *in vivo*, further emphasizing the need to regulate both pathways to induce indefinite survival of the graft.

## Abstract 2

Using a skin transplant model, Smyth et al observe that transference of MHC:peptide complexes between donor graft tissue and recipient dendritic cells occurs throughout the life span of the graft and contributes to anti‐graft CD8 T cell responses. See the editorial from Burlingham on page 5.

**Abbreviations**
cDC: conventional dendritic cell
DC: dendritic cell
DT: diphtheria toxin
sdLN: skin‐draining lymph node

## Introduction

The major obstacle to transplantation success is the recognition by the recipient immune system of donor major histocompatibility complex (MHC molecules) 1, 2. Two major pathways have been identified. The first is the “direct pathway” in which donor dendritic cells (DCs) present intact donor MHC–peptide complexes to recipient T cells. This pathway is characterized by a very high frequency of T cells specific for alloantigens 3, 4. The second, “indirect pathway” of allorecognition involves the presentation of peptides derived from donor MHC molecules by recipient DCs. Based on the fact that donor‐derived DCs are eliminated within days of transplantation, it has been argued that the indirect response represents the major threat to long‐term transplant survival, and is consequently the key target for regulation. However, since we proposed the existence of the third pathway of allorecognition, based on the evidence that intact MHC‐class I molecules can be transferred between DC subsets and between DCs and endothelial as well as epithelial cells, this assumption has been challenged 5, 6, 7, 8. The discovery of this new pathway of allorecognition suggests that the direct pathway could last for the life of the transplant and pose another challenge for the induction of long‐term transplant survival 6.

So far, the contribution of acquired MHC‐class I molecules has been assessed only at early time points. In both heart and kidney transplant settings, transfer of intact allo‐MHC to recipient DCs has been demonstrated at days 1–5 posttransplant for heart 9 and day 8 for kidney 10 in the heart transplant model the transferred MHC led to priming of both humoral and cellular alloimmunity 9.

In addition, the capacity of the transfer of donor‐MHC molecules to activate T cells with direct allospecificity has not been tested. Recently, Celli et al (2011) elegantly demonstrated, using intravital imaging, that following skin transplantation, donor‐derived dermal DCs migrated out of the transplant and that this occurs before recipient CD11b^+^ mononuclear cells, which consisted of neutrophils, mononuclear cells, and inflammatory DCs, arrived at the transplant site 11. Recipient CD11b^+^ cells acquired and presented alloantigen (via cross‐presentation) to CD8^+^ T cells in the skin‐draining lymph node (sdLN) 11. Given that donor dermal DCs leave the transplant and that host infiltrating CD11b^+^ cells return to the sdLN carrying antigen present in the transplant, in the above model, it is possible that alloantigen in the form of intact allo‐MHC class I is acquired in two different ways. Firstly, recipient resident DCs, present in the spleen and dLN, could acquire MHC‐class I from donor dermal DCs, or secondly recipient graft‐infiltrating cells by sampling the parenchymal cells could acquire MHC molecules and then traffic to the sdLN. Given that MHC‐class I is present throughout the life span of the graft, it is feasible that acquisition of allo‐MHC molecules by recipient DCs drives CD8^+^ T cell responses even at late time points after transplantation.

To test these hypotheses, MHC‐class I transfer was evaluated in indirect (also referred to as cross‐presentation or cross‐priming) pathway deficient recipient mice 12, in the absence and presence of donor‐derived DCs.

## Materials and Methods

### Mice

C57BL/6 (H‐2^b^) mice, 6–10 weeks of age were purchased from Harlan Olac (Bicester, UK). B6.CD11c‐GFP‐DTR mice (CD11c.DTR.GFP mice), Transgenic B6 mice expressing membrane‐bound chicken ovalbumin (Actin‐mOVA mice, a kind gift from Dr Randy Noelle), OT‐1Rag^−/−^ and H‐2K^Bm1^ mice were bred and maintained under sterile conditions. B6.K^d^ were a kind gift from Pat Bucy. B6.Batf3^−/−^ (Batf3^−/−^) mice were a kind gift from Dr Kenneth Murphy (Washington University School of Medicine) 12. Mouse handling and experimental procedures were conducted in accordance with the Home Office Animals Scientific Procedures Act of 1986.

### Cell culture medium

Cell cultures were performed in RPMI 1640 (Sigma, Poole, UK) medium supplemented with 100 IU/mL penicillin, 100 μg/mL streptomycin, 2 mM l‐glutamine, 0.01M *N*‐2‐hydroxyethylpiperazine‐*N*′‐2‐ethanesulfonic acid, 50 μM 2β‐mercaptoethanol (Thermo Fisher, Paisley, UK), and 10% heat‐inactivated fetal calf serum (FCS) (SERAQ, Sussex, UK). Cells were maintained at 37°C in a humidified atmosphere with 5% CO_2_.

### Antibodies and flow cytometry

All the mAbs used, unless stated otherwise, were purchased from Affymetrix eBiosciences, Cheshire, UK. Fluorochrome‐conjugated (fluorescein isothiocyanate, phycoerythrin [PE], allophycocyanin [APC]) mAbs against the following mouse cell‐surface antigens—CD11c, Vα2, and CD8—were used with their relevant isotype controls. For flow cytometry analysis, cells were labeled with fluorochrome‐conjugated mAbs for 30 min at 4°C, washed twice in FACS buffer (phosphate‐buffered saline/2% FCS/0.1% sodium azide), and analyzed on a FACSCalibur^™^, using the Cell Quest^™^ software (Becton Dickinson, Mountain View, CA). Subsequent off‐line analysis was performed with FlowJo software (Treestar, Ashland, OR).

### Purification and labeling of CD8^+^ T cells

Responder T cells were purified from red blood cell (RBC)–depleted splenocytes isolated from OT‐1 Rag^−/−^ mice. A single‐cell suspension was obtained by passing spleens and/or pooled lymph nodes through a 70‐μm cell strainer (Fisher Scientific, Loughborough, UK). Erythrocytes were lysed using ACK buffer (0.15M NH_4_Cl/1 mM KHCO_3_/0.1 mM Na_2_‐EDTA). The purity of responder T cells was consistently between 90% and 95%. T cells were labeled with 1 μM of Vybrant CFDA SE (CFSE (5) and ‐6)‐ Carboxyflourescein Diacetate, Succinimidyl Ester) (Thermo Fisher) according to the manufacturer's instructions before being injected.

### Skin grafting

Skin grafting was done according to the technique described by Billingham and Medawar 13 with some modifications. Full‐thickness donor tail skin (0.5–1 × 0.5–1 cm) was grafted on beds prepared on the right lateral flank of recipients. The graft site was protected by a waterproof plaster (Elastoplast, Birmingham, UK), which was removed on day 7. The grafts were observed every day afterwards and considered rejected when no viable skin remained. Graft survival between two groups was compared using the log‐rank test.

### MHC‐class I transfer experiments

Following transplantation, some recipient Batf3^−/−^ mice were injected i.v. with 200 μg of dsRNA (polyinosinic‐polycytidylic acid) to ensure a block in cross‐presentation in the presence or absence of 4 ng/g body weight of diphtheria toxin (DT) (Sigma‐Aldrich, Dorset, UK) via i.v. and i.p. injection to remove the GFP^hi^ DCs as described by Prlic et al 14. H‐2K^Bm1^ mice that received a transplant were injected with DT only. One day later, 4 × 10^6^ CFSE‐labeled T cells isolated from OT‐1Rag^−/−^ mice were adoptively transferred i.v. T cell proliferation was measured on day 3 following adoptive transfer of the labeled T cells via flow cytometry following staining with an anti‐Vα2‐PE antibody specific to the transgenic T cells and an anti‐CD8α‐APC.

Additionally, CD11c.DTR.GFP mice received a B6.K^d^ skin transplant, and 7 days later mice were treated with picryl chloride (2‐chloro‐1,3,5‐trinitrobenzene, Sigma Aldrich) for 4 days. Spleens were harvested and RBC‐depleted splenocytes were stained with antibodies to CD11c and K^d^. Expression of K^d^ on cells was measured by flow cytometry.

### T cell proliferation and cytokine assays

For *in vitro* studies, T cells from OT‐1 Rag^−/−^ mice were isolated using a CD8^+^ T cell isolation kit (Miltenyi Biotech, Surrey, UK). The purity of responder T cells was assessed using PE‐conjugated anti‐CD8 antibodies (clone 53‐6.7). The purity of T cells was consistently between 90% and 95%. CD11c selected DCs were isolated using a CD11c isolation kit (Miltenyi Biotech) following manufacturers’ instructions. 10^5^ purified CD8^+^ T cells and 10^5^ CD11c were stimulated in triplicate wells of a 96‐well plate. T cell proliferation was measured by [^3^H] thymidine incorporation after 3 days in culture. Results are shown as mean count per minute of triplicate determinations ± SD. To measure interferon‐γ (IFNγ) production, culture supernatant, taken from the above cultures, were analyzed using an IFNγ‐specific enzyme‐linked immunosorbent assay (ELISA) kit, following manufacturer's instructions (eBioscience). Results are shown as mean pg/mL of triplicate determinations ± SD.

### Statistical analysis

Data are represented as mean ± standard error of the mean where appropriate. Graft survival was depicted using Kaplan–Meier analysis and groups were compared by log‐rank (Mantel–Cox) testing. To determine statistical significance, a Student's t‐test (unpaired, two‐tailed) was carried out using the GraphPad Prism software, http://www.graphpad.com/prism/prism.htm. In the figures, p‐values <0.05 are indicated by *, p < 0.01 by **, and p < 0.001 by ***, whereas nonsignificant p‐values are labeled “ns.” Values of p < 0.05 were considered significant.

## Results

### mOVA‐expressing skin allografts are rejected in the absence of CD8α^+^ and CD103^+^ DCs

**Figure 1:**
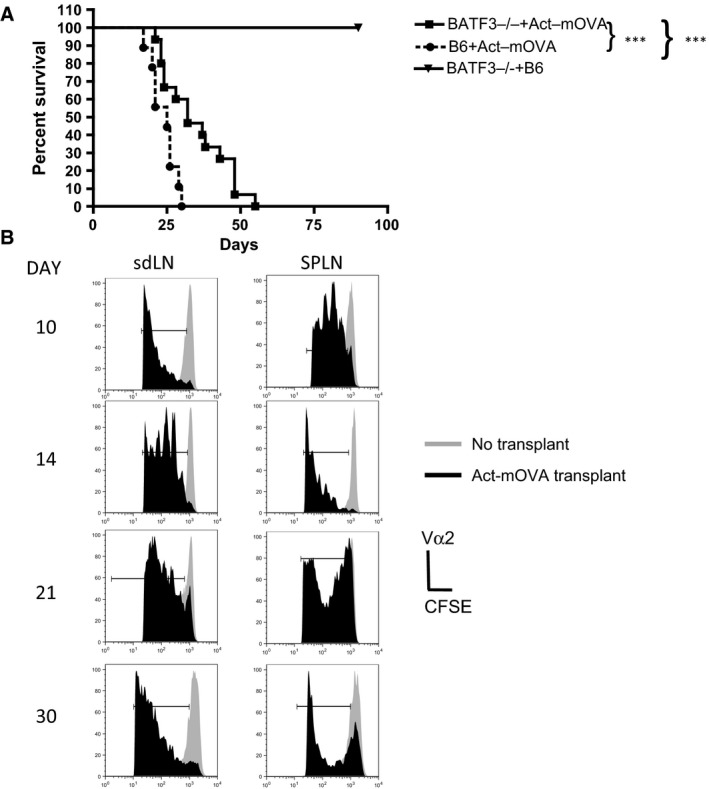
**Act‐** **mOVA** **skin grafts are rejected in the absence of cross‐presentation.** (A) Batf3^−/−^ mice received either an Act‐mOVA or a B6 skin transplant whereas B6 mice received only Act‐mOVA skin. Mice were monitored daily, and rejection was deemed as the day when no viable skin remained. ***p < 0.001 (Kaplan–Meier). Data are representative of three experiments with five mice per condition. (B) Batf3^−/−^ mice were injected with 4 × 10^6^ CFSE‐labeled OT‐1Rag^−/−^ T cells on days 10, 14, 21, or 30 post Act‐mOVA skin transplantation. Control mice received CFSE‐labeled T cells only (no transplant). After 3 days, proliferation of OVA‐specific T cells in the spleen (SPLN) and draining lymph nodes (dLNs) was measured. Cells were stained with antibodies to Vα2 and CD8 and CFSE dilution measured on Vα2^+^ CD8α^+^ only cells by flow cytometry. Panels represent the histogram profile of CFSE staining of T cells isolated for an individual mouse. Gray‐filled histograms represent the proliferation profile of OT‐1Rag^−/−^ T cells in control mice, whereas the black histograms represent the proliferation profile of OT‐1Rag^−/−^ T cells in Act‐mOVA skin transplant recipients. Data shown are representative of three experiments.

Rejection of skin expressing OVA, a single minor mismatch antigen, has previously been shown in B6 recipient mice 15. Injection of OVA‐specific CD8^+^ T cells, isolated from OT‐1 T cell receptor (TCR)–transgenic mice, into these transplanted B6 mice indicated the presence of OVA antigen in both sdLNs and spleen following skin transplantation 15. Activation of these T cells may be due to recognition of antigen in a variety of ways including antigen presented by donor DCs, direct recognition, or cross‐presentation by recipient DCs, or by recipient DCs presenting acquired MHC‐peptide complexes from the transplanted tissues. To assess the contribution of cross‐presentation in this model, we compared the rejection kinetics of Act‐mOVA skin in B6 mice and Batf3^−/−^ recipient mice (H‐2b). Batf3^−/−^ mice lack CD8α^+^ conventional DCs (cDCs), the DC subset regarded as the main cross‐presenters, as well as the nonlymphoid CD103^+^ migratory cDC population. In comparison to B6 mice, Batf3^−/−^ mice reject OVA skin transplants at a slower rate (mean survival time was 25 days on B6 recipients compared to 32 days on Batf3^−/−^ recipients, Figure 1A) suggesting that either, or both, the CD8α^+^ and the CD103 DC subset contribute to the rejection of skin transplants in the B6 mice. However, Act‐mOVA, but not control B6 skin transplants, were rejected by Batf3^−/−^ mice even in the absence of these cross‐presenting DC subsets (Figure 1A). Next we measured OVA‐specific CD8^+^ T cell response in the Batf3^−/−^ recipient mice receiving Act‐mOVA skin. Injection of CFSE‐labeled CD8^+^ T cells, isolated from OT‐1Rag^−/−^ mice, into transplanted mice on days 10, 14, 21, and 30 after transplantation resulted in T cell proliferation, measured 72 h later by CFSE dilution, at all time points (Figure 1B). The data therefore indicate that OVA antigen was present in the spleen and sdLN for a prolonged period (Figure 1B). Interestingly, even when there was very little skin left, day 30, posttransplant, T cell proliferation was still observed.

### MHC‐class I molecules are acquired throughout the life span of the transplanted skin

**Figure 2:**
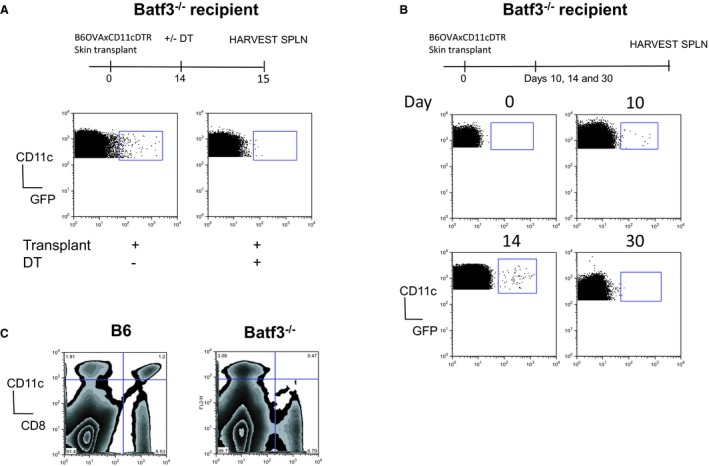
**Donor** **DC** **s present after transplantation can be removed following treatment with** **DT** **.** (A) Batf3^−/−^ mice received an Act‐mOVAxDTR/GFP skin transplant and 14 days later some mice received 4 ng/g body weight of DT. Control mice received no DT. After 24 h the presence of CD11c^+^ cells expressing GFP was assessed by flow cytometry. (B) Batf3^−/−^ mice received Act‐mOVAxDTR/GFP a skin transplant and the presence of CD11c^+^ cells expressing GFP was assessed by flow cytometry 10, 14, and 30 days posttransplant. Panels represent the dot‐plot profile of CD11c versus GFP expression on live cells for each individual mouse. (C) Spleens isolated from B6 (left) and Batf3^−/−^ (right) mice 14 days posttransplant were stained with antibodies to CD8 and CD11c. Panels represent the contour prolife of CD11c versus CD8 expression on live cells for each individual mouse. The data shown are representative of three experiments. DCs, dendritic cells; DT, diphtheria toxin; SPLN, spleen.

The aforementioned observations suggest that the presentation of OVA by either donor DCs or via the acquisition of intact MHC:peptide complexes by recipient DCs drives T cell proliferation following transplantation in the Batf3^−/−^ mice. To assess the contribution of MHC‐class I:OVA peptide complexes, acquired by recipient DCs, and to exclude the contribution of donor‐derived DCs directly presenting OVA peptide, to CD8^+^ T cells, Act‐mOVA‐expressing mice were crossed with CD11c.DTR.GFP mice. In doing so, we created a model in which donor DCs, presenting MHC‐class I molecules and OVA peptide, could be removed by using DT, following Act‐mOVAxDTR/GFP skin transplantation (Figure 2A). Although it has been reported that donor‐derived DCs are killed quickly following transplantation in a fully mismatched setting 16, donor‐derived DCs, as measured using CD11c^+^GFP^+^ expression, were observed in the spleen of recipient mice up to 14 days after transplantation. However, after 30 days donor DCs were completely absent (Figure 2B). No donor‐derived DCs were observed in the dLNs at any time point assessed (data not shown), suggesting that too few of these cells were present in this location in our experimental setup.

**Figure 3:**
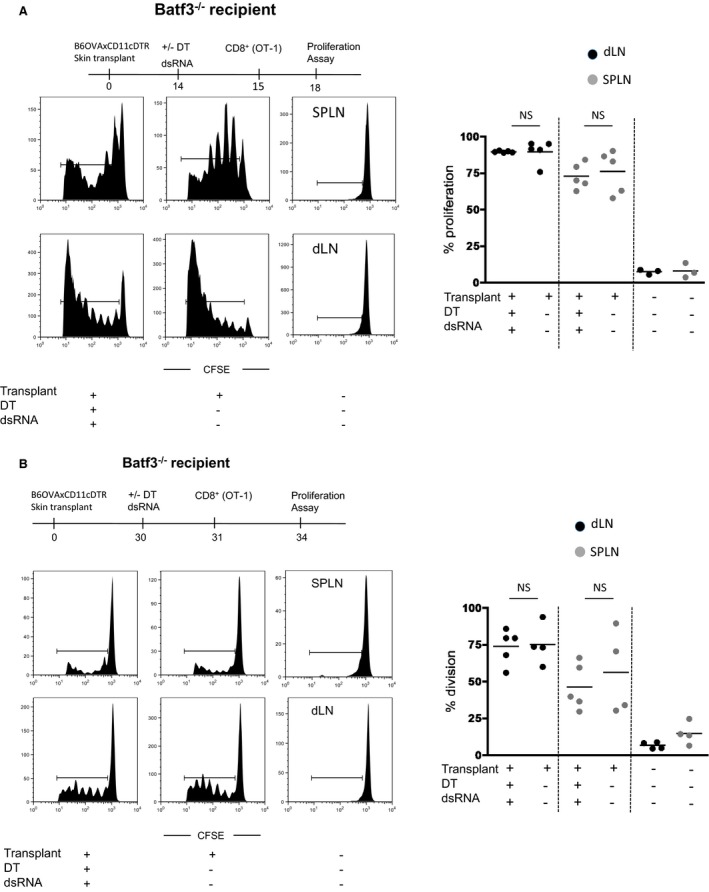
**Donor** **MHC** **‐class I molecules are acquired throughout the life span of the transplant and can induce** **CD** **8** ^**+**^ **T cell responses.** Batf3^−/−^ mice received an Act‐mOVAxDTR/GFP skin transplant and 14 (A) or 30 days (B) posttransplant; some mice received 4 ng/g of DT and 200 μg of dsRNA (left panels) 24 h prior to injection of CFSE‐labeled OT‐1Rag^−/−^ T cells. Control, transplanted mice received no DT (middle panels). After 3 days, proliferation of OT‐1Rag^−/−^ T cells, identified using antibodies to Vα2 and CD8, in the spleen (SPLN) and draining lymph node cells (dLNs) was measured by flow cytometry. Control mice received CFSE‐labeled OT‐1Rag^−/−^ T cells only (right panels). Histograms shown represent the proliferation of CD8α, Vα2, and CFSE‐positive cells for an individual mouse. The percentage proliferation of OT‐1Rag^−/−^ T cells, from five mice per group, is shown. Each dot represents an individual mouse. Statistical analysis using an unpaired t‐test with no significant difference denoted as NS. DT, diphtheria toxin.

To remove donor DCs, recipient Batf3^−/−^ mice were treated with DT, as previously published 8, 14 and 30 days post skin transplantation. It has been reported that under inflammatory conditions, CD8α^+^ DCs develop in the Baft3^−/−^ mice, although very few CD8α^+^ DCs were present in the spleens day 14 posttransplant (Figure 2C). However, to completely circumvent this possibility, we injected recipient mice with dsRNA intravenously as we, and others, have previously shown that this treatment results in the depletion of CD8α^+^ DCs 8. Importantly, when OVA‐specific OT‐1Rag^−/−^ T cells were injected 24 h, or 72 h (data not shown), after depletion of donor DCs using DT and dsRNA treatment, proliferation was observed in both the spleen and the sdLN of the recipient mice (Figures 3A and B). Importantly, no significant differences in the overall T cell responses were observed in the presence or absence of donor DCs. These findings suggest that at days 14 and 30 posttransplantation it was the recipient DCs that were presenting antigen to the T cells in this model. As expected, less T cell proliferation was observed over time due to a diminishing antigen load as the graft was rejected.

Taken together, the data suggest that the acquisition of MHC‐class I:peptide complexes from either donor parenchymal cells or dying donor DCs by recipient APCs drives antigen‐specific CD8^+^ T cell responses throughout the life span of the transplant.

### Recipient‐derived DCs acquire MHC‐class I molecules from donor cells

**Figure 4:**
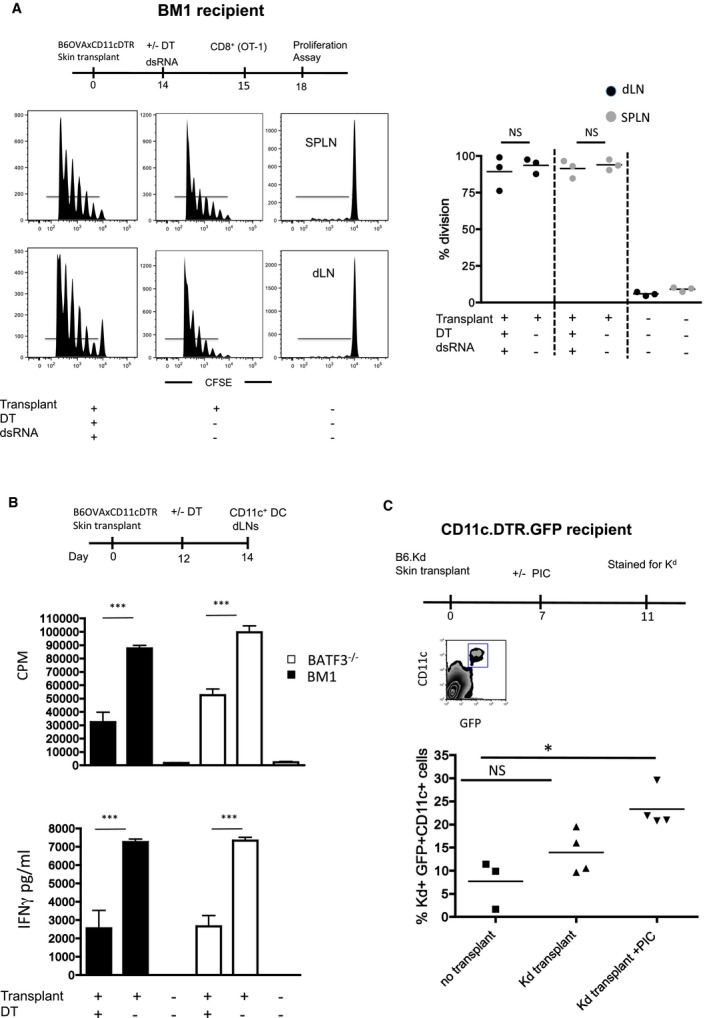
**Acquisition of donor** **MHC** **‐class I molecules by recipient** **DC** **s activates antigen‐specific T cells.** (A) H‐2K^B^ ^m1^ mice received an Act‐mOVAxDTR/GFP skin transplant and 14 posttransplant mice received 4 ng/g of DT (left panels) 24 h prior to injection of CFSE‐labeled OT‐1Rag^−/−^ T cells. Control, transplanted mice received no DT (middle panels). After 3 days, proliferation of OT‐1Rag^−/−^ T cells, identified using antibodies to Vα2 and CD8, in the spleen (SPLN) and draining lymph node cells (LNs) was measured by flow cytometry. Control mice received CFSE‐labeled OT‐1Rag^−/−^ T cells only (right panels). Histograms shown represent the proliferation of CD8α, Vα2, and CFSE‐positive cells for an individual mouse. The percentage proliferation of OT‐1Rag^−/−^ T cells, from three mice per group, is shown. Each dot represents an individual mouse. Statistical analysis using an unpaired t‐test with no significant difference is denoted as NS. (B) H‐2K^B^ ^m1^ (Bm1, black column) and Batf3^−/−^ (white column) mice received an Act‐mOVAxDTR/GFP skin transplant and 12 days later some mice received 4 ng/g of DT. Forty‐eight hours following DT injection, dLNs were harvested and CD11c^+^ DCs were isolated. CD11c^+^ DCs were used to stimulate OT‐1 T cells at a 1:1 ratio *in vitro* and proliferation was measured on day 3 using ^3^H incorporation. Control CD11c^+^ DCs were isolated from unmanipulated mice. Data shown represent a pool of three individual experiments and mean (CPM) ± 1 SEM are shown. IFNγ present in culture supernatant was measured using a specific ELISA and is expressed in pg/mL. Data shown represent a pool of three individual experiments and mean (CPM) ± 1 SEM are shown. Statistical analysis using an unpaired t‐test with significance denoted as p < 0.001 by ***. No significant difference is denoted as NS. (C) CD11c.DTR.GFP mice received a B6.K^d^ skin transplant and 7 days later some mice received a picryl chloride application to the grafted tissue. Four days following treatment, RBC‐depleted splenocytes were stained with antibodies to CD11c and K^d^. Splenocytes from nontransplanted and nontreated mice were used as controls. The data represent the profile of K^d^ expression on viable CD11c^+^ GFP ^+^ cells. Statistical analysis using an unpaired t‐test with significance denoted as p = 0.038 by *. No significant difference is denoted as NS. CPM, counts per minute; DCs, dendritic cells; DT, diphtheria toxin; ELISA, enzyme‐linked immunosorbent assay; IFNγ, interferon γ; RBC, red blood cell.

To confirm that donor MHC‐class I molecules, present on graft tissue, were acquired by recipient DCs and presented to T cells in the dLNs, we repeated the Act‐mOVAxDTR/GFP skin transplants using H‐2K^Bm1^ recipient mice. We have previously shown that DCs isolated from H‐2K^Bm1^ do not cross‐present either soluble or cell‐associated OVA to OT‐1 T cells 1, 8. However, these DCs can present acquired MHC:OVA complexes to OT‐1Rag^−/−^ T cells 1, 8. Transplanted mice were treated with DT 14 days after transplantation, control mice received no DT, and 1 day later mice received CFSE‐labeled OT‐1 T cells. As expected from our previous observations in the Baft3^−/−^ recipients, no significant differences in the overall T cell responses were observed in the presence or absence of donor DCs (Figure 4A). These findings confirm that in the absence of cross‐presentation, recipient DCs acquire donor MHC:peptide and activate CD8^+^ T cells *in vivo*. This was further confirmed *in vitro*. DT treatment in transplanted mice (both Batf3^−/−^ and H‐2K^Bm1^) was performed at day 12, to remove donor DCs, and 2 days later CD11c^+^ DCs were isolated from dLNs and co‐cultured with T cells isolated from OT‐1Rag^−/−^ mice. T cell proliferation and cytokine production (IFNγ) were measured *in vitro* after 3 days of co‐culture (Figure 4B). DCs isolated from the dLNs of both Batf3^−/−^ and H‐2K^Bm1^ mice treated with DT were capable of activating OVA‐specific T cells to proliferate and produce IFNγ as compared to DCs isolated from nontransplanted mice (Figure 4B). However, the overall response was less than that seen with transplanted mice not treated with DT, where DCs presenting antigen both directly (donor derived) and via acquisition (recipient DCs) were present. These data confirm that recipient DCs were capable of acquiring MHC:OVA complexes from transplanted tissue and, together with direct presentation of antigen from donor DCs, activates antigen‐specific CD8^+^ T cells.

Having established that donor MHC‐class I transfer is functional on recipient DCs and drives CD8^+^ T cell activation, we then directly measured the transfer of MHC‐class I molecules and addressed whether inflammation influences this phenomenon. To do so, skin transplants from B6.K^d^ transgenic mice (B6 mice expressing H‐2K^d^) into CD11c.DTR.GFP recipient mice were performed and the K^d^ expression on GFP^+ve^ CD11c^+^ cells was measured by flow cytometry following staining with anti‐K^d^ antibodies. As expected, we found that recipient GFP^+^CD11c^+^ DCs, present in the spleen, acquired K^d^ (Figure 4B, middle panel) following transplantation, with an increase in the number of recipient DCs expressing K^d^ being observed (3.74–16.2%) when the skin irritant picryl chloride was administered to the transplanted K^d^ skin (Figure 4C, last panel). We conclude that during transplantation recipient DCs can acquire intact allo‐MHC‐class I molecules from graft parenchyma and that under inflammatory conditions this acquisition is enhanced.

## Discussion

In this study we have demonstrated, for the first time, that following transplantation, donor MHC‐class I acquisition by recipient DCs occurs throughout the life span of the transplant. We observed, using an OVA antigen skin transplant model, that acquired MHC‐class I:OVA complexes on recipient DCs were the main source of antigen capable of inducing antigen‐specific CD8^+^ T cells. In addition, we provide evidence for the first time that inflammation of the graft contributes to the transfer of MHC‐class I molecules using K^d^ skin as model antigen. Taken together, the data presented here argue that the direct pathway, as a consequence of acquisition of MHC class I, together with the indirect pathway are major drivers of alloimmunity and should be considered when designing tolerance‐promoting protocols.

In this study, we have shown that removing donor DCs in both the Batf3^−/−^ and H‐2K^Bm1^ *in vivo* skin models did not substantially reduce antigen‐specific *in vivo* T cell proliferation, suggesting that donor DCs may not monopolize initiation of the direct alloresponse. It should be noted that in our model, DT injection depletes mostly dermal DCs, with minimal effect on epidermal Langerhans cells. The lack of an essential role for donor DCs in transplant rejection has been shown by others using several murine transplantation models including heart, skin, and kidney. One example was highlighted using a model similar to ours. Garrod et al made use of the CD11c.DTR mice to selectively deplete donor DCs in heart allografts using DT 16. These authors did not report a delay in rejection by removing donor DCs, suggesting that donor DCs, in contrast to what has previously been reported, are not essential for initiating alloimmune responses.

As discussed in the Introduction, intact allo‐MHC‐class I may be acquired by several different routes, including delivery of these molecules by skin donor dermal DCs to recipient resident DCs. It has been reported that donor DCs that migrate out of transplanted organs, in a fully mismatched setting, are quickly killed by natural killer cells in the secondary lymphoid tissues of the recipient 16. In the skin model described here, donor DCs are present up to 14 days posttransplant, suggesting that acquisition of MHC class I by resident recipient DCs from these donor cells may occur and contribute to the rejection of OVA skin transplants. It is also possible that recipient B cells also acquire donor MHC class, and this warrants further investigation.

Depleting recipient DCs has been shown to significantly prolong graft survival 16. Our transplant data suggest that this may be due to the removal of recipient DCs that have acquired donor MHC‐class I molecules. Our data seem to be in contrast to the work presented by Kurts et al 17. These authors showed that when TCR‐transgenic OT‐1 T cells, specific for an OVA peptide presented by H‐2K^b^, were injected into mice transgenic for OVA expressed in the pancreas, the transferred T cells divided vigorously in the draining lymph node. This was presumed to result from the capture and processing of OVA by trafficking DCs. However, if the OVA‐transgenic mice were made chimeric with H‐2K^Bm1^ bone marrow (K^Bm1^ cannot present the OVA peptide to OT‐1 T cells), no OT‐1 T cell division was seen. This suggested that the trafficking of K^Bm1^ expressing DCs did not acquire intact complexes of K^b^ with OVA peptides from the pancreatic β cells in sufficient quantities to induce OT‐1 T cell proliferation 17. This observation led to the idea that MHC‐class I transfer *in vivo* may be more efficient under inflammatory conditions as compared with the steady state. Indeed, we demonstrate the importance of inflammation by using the skin irritant picryl chloride. This treatment led to a fivefold increase in acquired MHC‐class I molecules in a skin transplant setting, further extending our previous findings 5. As immunosuppressive drugs are used extensively following organ transplantation, testing whether MHC transfer occurs under these conditions will help elucidate the role of the semidirect pathway in solid organ rejection.

An important question that has not yet been addressed is how the transfer of MHC‐class I:peptide complexes occurs *in vivo*. Indeed, whether the transfer occurs directly via cell‐to‐cell interaction including membrane “nibbling” 18, 19 or through production of either apoptotic bodies or exosomes warrants further investigation 20, 21. Whether specific receptors are involved in the uptake of MHC:peptide complexes and whether inflammation influences any of the aforementioned mechanisms are questions that need further investigation. Although bone marrow–DCs have been shown to produce exosomes containing MHC‐class I molecules 22, 23, Wakim and Bevan observed using an *in vitro* transwell system that direct contact, rather than exosomes release and acquisition, was important for the transfer of MHC‐class I molecules 24. However, given that this experimental system may limit exosome transfer, the question of whether exosomes or apoptotic bodies released from donor DCs or parenchymal cells *in situ* contribute to the semidirect pathway is something that warrants further investigation. This has recently been addressed with respect to donor DCs 25.

In summary, we have demonstrated for the first time that MHC transfer lasts at least for the life of the transplant, activating CD8^+^ T cells with specificity against MHC‐class I:peptide complexes transferred from the graft to recipient antigen presenting cells for the duration of the graft. Furthermore, we have proven that inflammation of the graft further increased the amount of MHC transfer. Altogether, the data presented here extend our previous observations, further emphasizing that both the indirect and the direct pathway of allorecognition are likely to persist for the life of a transplant. Consequently, both pathways need to be regulated if transplantation tolerance is to be achieved.

## Disclaimer

The views expressed are those of the author(s) and not necessarily those of the NHS, the NIHR, or the Department of Health.

## Disclosure

The authors of this manuscript have no conflicts of interest to disclose as described by the *American Journal of Transplantation*.
